# Management and Outcomes for Peritoneal Dialysis Patients Diagnosed with Abdominal Hernias

**DOI:** 10.3390/life14081003

**Published:** 2024-08-13

**Authors:** Cristian Iorga, Cristina Raluca Iorga, Iuliana Andreiana, Simona Hildegard Stancu, Iustinian Bengulescu, Victor Strambu

**Affiliations:** 1 Faculty of Medicine, “Carol Davila” University of Medicine and Pharmacy, 050474 Bucharest, Romania; cris.iorga@yahoo.com (C.I.); simonastancu2003@yahoo.com (S.H.S.); iustinian.bengulescu@umfcd.ro (I.B.); drstrambu@gmail.com (V.S.); 2 Surgery Clinic, “Dr. Carol Davila” Clinical Nephrology Hospital, 010731 Bucharest, Romania; 3 Nephrology Clinic, “Dr. Carol Davila” Clinical Nephrology Hospital, 010731 Bucharest, Romania

**Keywords:** peritoneal dialysis, abdominal wall hernia, hernia repair

## Abstract

Background/Objectives: The success of peritoneal dialysis is highly dependent on the integrity of the abdominal wall. Therefore, routine examination and treatment of abdominal hernias can prevent peritoneal dialysis (PD) failure, discontinuation, and conversion to hemodialysis. In this present study, we present our examination protocol for patients proposed for PD and our attitude in treating parietal defects in patients on peritoneal dialysis. Objectives: highlight whether PD is a risk factor for the occurrence of ventral hernias, the relationship between associated pathologies and the occurrence of hernias and the need for an HD switch in the postoperative period. Methods: Between January 2016 and December 2022, a group of 133 patients proposed for insertion of a PD catheter were evaluated according to the protocol established by our hospital. Routine examination for the diagnosis of abdominal hernias and repair before starting the DP is part of the procedure. We included patients with a 3 year minimum follow-up after insertion and evaluated the incidence of parietal defects that appeared during PD treatment. Results: Nine patients were diagnosed and operated on for abdominal hernia before starting peritoneal dialysis and none of them had a recurrence of hernia during PD. Twelve patients were diagnosed with abdominal hernias during dialysis treatment (9% incidence) and the median length of time at which parietal defects occur during PD is 12.5 months [range 2–48]. Median BMI is 27.12 [range 22.3–31.24], with a female–male ratio of 2:1 Five patients were transferred to HD, three permanently and two patients temporarily. No patient abandoned PD treatment due to the presence of an abdominal parietal defect. Conclusions: Diagnosis of ventral hernias prior to the time of catheterization for PD leads to a decrease in the incidence of parietal defects during PD and is mandatory in patients who are candidates for PD. Open alloplastic surgical procedures are safe procedures with a low recurrence rate in PD patients. The postoperative continuation of PD is feasible but the decision is to be made by the multidisciplinary team and individualized for each patient.

## 1. Introduction

Peritoneal dialysis (PD) is an effective method of dialysis for patients with end-stage renal disease (ESRD) that has many advantages over hemodialysis (HD). Among these are the preservation of residual urinary function, independence from a dialysis center, continuation of a normal social life, possibility of remote monitoring, lower cost compared to HD, and possibility of immediate isolation, as happened in the COVID-19 pandemic [1,2,3,4,5,6].

In Romania, the latest centralized data of the Romanian Renal Registry (RRR) shows a total of 17,500 patients in HD and 255 patients in PD. Data reported by the United States Renal Data System (USRDS) shows a preponderance of HD over PD, with the “champions” of PD being El Salvador with 64.8% and Hong Kong with 45.6% of the total number of patients on dialysis [7,8].

The COVID-19 pandemic produced a worldwide increase in the number of peritoneal dialysis patients compared to hemodialysis patients, with the explanation being the high number of SARS-Cov2 infections in dialysis centers. Also, in Romania in 2020, there was a 60% increase in the number of patients in PD, followed by a decrease in 2021, and currently, the number of patients in PD is about the same as in the pre-pandemic period. In order to increase the number of patients with PD, the advantages of this method of dialysis should be popularized among patients and medical staff [7,9].

The long-term functionality of PD is a goal to which several factors contribute—the first step is the correct placement of the PD catheter, regardless of the insertion method chosen (laparoscopic, open surgery, peritoneoscopic) and regardless of the physician performing the insertion (surgeon, nephrologist, interventional radiologist) [10,11].

In Romania, the first catheterization for PD was performed in 1995 by the open surgical method. Currently, catheterization for PD is performed both open and laparoscopically [12].

The advantages of laparoscopic peritoneal dialysis catheter placement are extensively discussed in the literature and one of the main advantages is the visualization of the entire peritoneal cavity and the possibility of revealing incipient hernias that cannot be diagnosed by clinical examination or imagistic methods [13,14,15].

Studies from recent years indicate that PD is a risk factor for an increased incidence of ventral hernias compared to the general population. The mechanisms involved are associated with the duration of PD, polycystic kidney disease, and the volume of dialysis solution used correlated with increased intra-abdominal pressure, increased body mass index (BMI), and previous surgery [16,17].

Early diagnosis of the occurrence of ventral hernias in PD patients is very important to ensure their surgical treatment as quickly as possible, thus preventing the occurrence of hernia-related complications (incarceration, strangulation) or PD-related complications (genital edema, leakage, PD inefficiency, and finally, PD failure) [18,19,20].

Currently, there is no common opinion about the timing of parietal defect repair relative to the time of PD catheter insertion. There are three streams in the literature: surgery before catheter insertion, repair of the defect at the same time as catheter placement, and repair remotely after catheter placement, either immediately after diagnosis or when the patient becomes symptomatic for ventral herniation [21,22].

The first option seems to be increasingly used, especially in patients who do not represent an urgency for peritoneal dialysis entry, to allow the correct postoperative healing and to avoid the risk of recurrence. Surgical consultation performed a few months before PD entry, accompanied or not by imaging investigations (ultrasonography, computed tomography) if needed, is mandatory for the diagnosis of parietal defects, as suggested by ISPD (2019) and SAGES (2023) guidelines [21,23,24].

The option of repairing the parietal defect at the same time as the peritoneal dialysis catheter insertion is the ideal method, especially if the catheter is laparoscopically inserted, thus diagnosing incipient hernias [25,26,27,28,29,30].

The repair of the parietal defect occurring in the PD immediately after diagnosis is varied, supported by most studies [30,31,32].

Very few studies recommend repair of the previously diagnosed and unoperated parietal defect during PD when the hernia becomes symptomatic [21,31].

Surgical treatment of parietal defects in the general population is accomplished by classic open or laparoscopic techniques. Historical tissue-based procedures are practically abandoned at present and reserved only for particular situations [33].

Laparoscopy is gaining more and more ground in comparison with open procedures, with two major advantages: shorter postoperative recovery and a low risk of recurrence. For inguinal hernias, two laparoscopic techniques are preferred: TAPP (TransAbdominal Preperitoneal) and TEP (Totally ExtraPeritoenal), and for umbilical hernia or other parietal defects, laparoscopic intraperitoneal placement of a dual mesh is used [34,35].

Currently, all open surgical procedures for parietal defects use alloplastic (mesh) materials. For open inguinal hernias, the Lichtenstein procedure is the most commonly used technique, and for umbilical hernia or other ventral parietal defects, the mesh is placed in various ways adapted to each patient [33,34,35,36,37].

In PD patients, most studies indicate surgery for parietal defects by an open surgical approach. There are very few studies discussing laparoscopic repair of parietal defects in PD patients. For inguinal hernias by TAPP or TEP procedures, the mesh is placed preperitoneal, which may lead to fibrosis of the dissected peritoneum during the healing process and degradation of the peritoneal epithelium, leading to a decrease in the efficiency of PD. For the other parietal defects in which laparoscopic intraperitoneal dual mesh is inserted, the discussion is related to the possibility of intraperitoneal adhesions and mesh infection if the patient develops peritonitis during PD [38,39,40,41].

The most common ventral hernias occurring in patients in PD are inguinal hernias, umbilical hernias, femoral hernias, pericatheter hernias, and incisional hernias in patients who have had previous abdominal surgery, in that order [20,21,30].

Early diagnosis of ventral hernias in patients undergoing PD is very important in order to ensure their surgical treatment as quickly as possible, thus preventing the occurrence of hernia-related complications (incarceration, strangulation) or PD-related inefficiency and, ultimately, PD failure [16,18].

## 2. Materials and Methods

We conducted a retrospective study on 133 patients in PD admitted at the Nephrology Hospital Dr. Carol Davila (Bucharest, Romania), a tertiary center of surgery in the treatment of ESRD patients between January 2016 and December 2022.

The study was performed in accordance with the Declaration of Helsinki and approved by the local Ethics Committee (no. 69, March 2024, Local Ethics Committee Dr. Carol Davila Teaching Nephrology Hospital Bucharest).

The study included all patients who were referred to our clinic for insertion of a PD catheter, or patients in PD who were referred for treatment of a ventral hernia. All patients were followed up at least 3 years after insertion of the respective catheter or after surgical cure of the hernia in order to detect postoperative recurrences. Exclusion criteria were aged <18 years, patients with acute renal failure, and missing data.

The objective of the study is to highlight whether PD is a risk factor for the occurrence of ventral hernias, the relationship between associated pathologies and the occurrence of hernias, and the need for an HD switch in the postoperative period.

All the data were retrospectively extracted from the electronic medical records, from the operation registers, and from PD treatment monitoring files.

### 2.1. Preoperative Evaluation of Patients Candidates for PD

The hospital protocol for patients who are candidates and wish to enter the PD program includes surgical consultation that is performed at least 3 months prior to catheter insertion. The main objectives of the surgical consultation are to highlight the presence of ventral hernias in patients who have undergone previous surgery to assess the integrity of the abdominal wall. If hernias or other parietal defects are diagnosed, patients benefit from surgical cure at least 2 months prior to the insertion of the PD catheter.

The clinical examination for the diagnosis of ventral parietal defects consists of an examination in the orthostatism and in the dorsal decubitus of all ventral hernia points (umbilical, inguinal, femoral, and Spigelian), and the area with the operative incision in patients who have had prior open surgery. We also carefully examine the incisions where trocars have been placed in patients who have undergone previous laparoscopic surgery. In obese patients, if the clinical examination is inconclusive due to a rich fat panniculus, we perform soft tissue ultrasonography.

In the case of inguinal hernia diagnosis, we adopt the Lichtenstein tension-free technique. The surgical technique consists of entering the inguinal canal, isolating the spermatic funiculus/round ligament, and treating the hernial sac. After resection of the hernial sac, the posterior wall and deep orifice are reconstructed. For this purpose, a 10/6 cm polypropylene mesh is fitted overlying the pubic tubercle by 2 cm and secured with polypropylene 2/0. After that, a drainage tube is inserted and the skin is sutured. 

In cases of umbilical hernias, we perform omphalectomy, resection, and treatment of the contents of the hernia sac. Afterward, we prepare the aponeurotic edges and suture them with Prolene 1 thread. Then, we fix a polypropylene mesh with a diameter that exceeds the defect by at least 5 cm in all directions and fix it with a non-absorbable Prolene 2/0 thread or with a medical adhesive (Glubran).

### 2.2. PD Catheter Placement

Insertion of PD catheters is performed laparoscopically, except for patients who have a contraindication for general anesthesia. One of the main operative times is also the thorough exploration of the peritoneal cavity to identify intraperitoneal adhesions and the exploration of hernia points (inguinal, femoral, umbilical, Spiegelian) to highlight possible incipient hernias.

Surgery for laparoscopic insertion of catheters for PD is performed under general anesthesia with an indwelling urinary catheter, which is removed at the end of the operation.

We use a 10 mm supraumbilical optical trocar and a 5 mm working trocar in the right flank. We then tunnel the rectus abdominis muscle on the left side in an oblique direction towards the bottom of the Douglas pouch, with a 10 mm trocar used for insertion of the catheter. After the correct positioning of the catheter at the bottom of the Douglas sac, it is tightened with polypropylene thread 3/0 at the point of externalization in the peritoneum with a fascial closure needle. The functionality of the catheter is checked by instillation and aspiration of 0.9% saline solution. 

The exit site of the catheter is placed at 2 cm from the superficial cuff through a minimum incision corresponding to the diameter of the catheter. 

The suture of the supraumbilical wound aponeurosis is realized with polypropylene thread no. 1.

Before surgery, we ask patients to administer a dose of sodium picosulphate to empty the digestive tract.

A single dose of antibiotic (Cefuroxime 1.5 g) is given at anesthetic induction.

### 2.3. Patients in DP Treatment

Surgical interventions for the cure of hernias are performed by open surgery under spinal anesthesia, with the insertion of a urinary catheter that is removed at the end of the operation and the administration of a prophylactic dose of antibiotic.

The routine evaluation of patients includes a pre-anesthetic consultation, the discontinuation of peritoneal dialysis 24 h before surgery, and peritoneal fluid drainage.

For the surgical treatment of inguinal hernias, we use the Lichtenstein tension-free procedure (described above), for umbilical hernias, we perform omphalectomy with the use of a supra-aponeurotic mesh, and for pericatheter hernias, after catheter extraction and suture of the aponeurosis, we use the supra-aponeurotic mesh. The surgical intervention ends with the placement of a supra-aponeurotic drainage tube, when necessary, a decision that depends on the extent of the dissection and the level of intraoperative blood loss. The same surgical protocol is used for patients diagnosed with parietal defects prior to PD catheter insertion.

In the case of bilateral inguinal hernias, we use the Lichtenstein technique for the surgical cure of both hernias.

Also, for all the surgeries described, we ask patients to stop antiaggregant therapy at least 7 days prior to surgery and to replace oral anticoagulants with low molecular weight heparin once a day for seven days preoperatively. 

For the postoperative management of patients, we prescribe fasting until bowel transit is resumed, parenteral rehydration, and the monitoring of vital parameters. Additionally, local compression is applied to the affected area to prevent bleeding or seroma formation. All patients (with or without a conversion to HD) are gradually transitioned back to their peritoneal dialysis regimen via small-dose intermittent shifts.

Statistical analysis was performed using the Analyse-it™ Standard Edition package (Analyse-it 4.80 Software, Ltd., Leeds, UK). Categorical variables are presented as percentages, with comparisons performed using Pearson’s χ2 test. Continuous variables are presented as mean with a 95% confidence interval (95% CI) or median and quartiles [1; 3], depending on their distribution. Microsoft Excel 2013 was used for graphs and tables.

## 3. Results

A total of 133 patients who met the inclusion and exclusion criteria were included in the study, with a mean age in the seventh decade, 64% male gender. Of these, in 126 patients, the PD catheter was inserted laparoscopically, and in 7 patients, it was inserted during open surgery. Out of the 126 patients with laparoscopic catheter insertion, 12 patients had previous abdominal surgeries (Table 1).

Through the clinical surgical examination protocol, a total of nine parietal defects were diagnosed: six male patients with inguinal hernias (one patient with a bilateral hernia) and three female patients with umbilical hernias.

The median BMI in men with an inguinal hernia was 25.12 [ranging from 22.43 to 28.34], and in women, it was 27.56 [ranging from 25.34 to 30.12].

In Table 2, we described the gender distribution and the cause of ESRD in these patients, with the remark that the men with bilateral inguinal hernias were diagnosed with polycystic kidney disease.

All patients underwent open surgery by Lichtenstein tension-free alloplastic technique/omphalectomy and placement of reinforcement mesh, with a minimum of 2 months before placement of the PD catheter.

Postoperatively, three complications occurred (33%) and were managed conservatively with per primam healing. It should be mentioned that the hematoma occurred in the patient with a bilateral inguinal hernia and was present on one side only (Table 2). We had no perioperative mortality.

On laparoscopic exploration of the peritoneal cavity at the time of catheter placement for PD, no incipient parietal defects requiring surgical cure were evident and no recurrence of hernias was encountered.

During PD, 12 parietal defects were diagnosed (9%), of which there was one inguinal hernia, eight umbilical hernias, two supraumbilical hernias, and one pericatheter hernia. None of these patients has had previous surgery for a parietal defect, so we cannot speak of a recurrence of hernias operated before catheter insertion. The gender ratio for female–male is 2:1 (eight females and four males). Diabetic and ischemic nephropathy are prevalent (about 60%) among ESRD causes. The median length of time at which parietal defects occurred during PD is 12.5 months [ranging from 2 to 48] and the median BMI is 27.12 [ranging from 22.23 to 31.24] (Table 3).

Patients with previous surgery had no parietal defects at the level of the anterior incisions. All parietal defects occurred in patients in the laparoscopic PD catheter insertion group except for one patient in the open insertion group. One patient represented an emergency for a strangulated supraumbilical hernia. Supraumbilical parietal defects are parietal defects of the incision for insertion of the optic trocar during laparoscopic placement. Five postoperative complications were recorded (42%) (Table 4).

Postoperatively, five patients were transferred to HD, out of which three patients were permanently transferred to HD and 2 patients were temporarily transferred to HD, with the rest of the patients continuing PD postoperatively.

Except for the patients who were permanently transferred to HD, the rest of the patients continued PD, and there were no patients who abandoned PD due to the occurrence of parietal defects.

In the postoperative follow-up period, none of the patients who continued PD developed a recurrence of parietal defects.

Patients are discharged 5–7 days after surgery, and the lavage of the peritoneal cavity is initiated on Day Two after surgery. It is performed twice a week during the first week with a volume of about 200 mL of solution. Then, the volume of the solution and the number of daily shifts are progressively increased for the next three weeks. From the fourth week, the patient returns to the pre-operative dialysis program.

## 4. Discussion

The incidence of inguinal hernia in the general population is about 27%, and of umbilical hernia about 16%, with inguinal hernia being more common in males than umbilical hernia, which is more common in females. The main causes of ventral hernias are BMI > 30, chronic obstructive pulmonary disease, chronic constipation, prostate adenoma, and patients who perform strenuous physical exertion daily [42,43,44].

The incidence of ventral hernias in PD patients is about 27%, with a higher incidence of umbilical hernia in males, exactly opposite to the general population. The majority of studies indicate an increased incidence of ventral hernias in PD patients, the causes being primarily increased intra-abdominal pressure at dialysis fluid introduction, male gender, polycystic renal or hepato-renal disease, and high BMI, although there are also studies that identify more frequent occurrence of hernias in patients with low muscle mass [16,17,44]. In our group, polycystic kidney or hepatorenal disease had an incidence of 25%, with the highest incidence in patients with chronic glomerulonephritis (50%).

In our study group, 9% of patients developed ventral hernias during peritoneal dialysis, of whom most were women (67%). We obtained this low incidence due to the hospital protocol whereby patients who are about to enter PD are surgically examined for ventral hernias; thus, 7% of patients with ventral hernias were found. Another important aid in early ventral hernia repair is the laparoscopic fitting of PD catheters, thus diagnosing early hernias that may escape previous clinical and imagistic examination. The discovery of incipient hernias is one of the reasons why we prefer laparoscopic insertion of PD catheters (95%) [21,23,45].

All patients included in the study, who were diagnosed prior to entering PD with ventral hernias (7%), underwent surgical cure of the parietal defect before PD catheter placement and, subsequently, did not have a recurrence of the parietal defect during PD.

For the laparoscopic technique, we used 10 mm and 5 mm trocars, but some authors recommend the use of 7 mm trocars for the insertion of the PD catheter and a 5 mm trocar with a 5 mm optic for the supraumbilical incision to prevent pericatheter and optic trocar hernias. Unquestionably, these aspects are important, but they are technical aspects that are related to the equipment of each hospital [13,14,15].

In our study, we did not diagnose any patients with incipient hernia during laparoscopic PD catheterization. If we had detected a hernia during laparoscopic exploration of the peritoneal cavity, our option would have been hernia repair in the same operative act as the open Lichtenstein technique, as indicated in the ISPD and SAGES guidelines [21,23,24].

Decreasing the incidence of parietal defects in patients with PD is the common goal of both the nephrologist and the surgeon. Both the clinical examination for the discovery of ventral parietal defects before the onset of PD and laparoscopic catheterization, which through the visual examination of the entire peritoneal cavity can reveal incipient parietal defects, contribute to this goal [24,25].

We consider the option of surgery during PD in patients diagnosed prior to PD and who become symptomatic to be unfeasible because it exposes the patient to risks related to PD (inefficiency of PD, possibility of sequestering the dialysis fluid in the hernia sac) and also related to the parietal defect (incarceration, strangulation, formation of intraperitoneal adhesions). This variant can be used as a backup method only for patients presenting high anesthetic risk (ASA score > 3) [46].

In all patients in the study, we used the alloplastic open surgical technique, both in patients in whom surgery was performed prior to insertion of the PD catheter (9 patients) and in patients who developed parietal defects during PD (12 patients). Postoperatively, eight complications occurred in 21 patients, which were managed conservatively with per primam healing. No recurrences of parietal defects were detected during postoperative follow-up. Although, in the general population, we predominantly use laparoscopic procedures for the surgical treatment of abdominal hernias, the lack of guidelines and few studies in this regard for patients with PD leads us to use alloplastic surgical procedures exclusively for these patients [47,48,49].

Only one patient presented pericatheter herniation in the second month after catheter insertion; they presented pericatheter leakage spontaneously resolved at 4 weeks after catheter insertion. Being a small defect (sliding of the hernia sac) with a correct functionality of the PD, surgery was performed 9 months after catheter insertion when the parietal defect started to increase in size.

A controversial discussion in the literature is related to the postoperative continuation of PD or the need to switch on HD and for how long. The main discussion is centered on the fact that PD, by increasing the intra-abdominal pressure during shifts, may predispose to an increased incidence of postoperative recurrences [28,30].

Of the 12 patients, 5 patients were switched on HD, of which 3 were permanent and 2 were temporary (one for 3 weeks and another 4 weeks). The definitive transition to HD in the three patients was carried out for major surgery in one patient (supraumbilical hernia with a strangulated loop, in which a segmental enterectomy was also performed); one patient who had a decrease in the efficiency of PD before surgery (patient in year 4 of PD), and one patient who did not want to continue PD. The rate of PD dropout and permanent transfer to HD due to parietal defects occurring during PD is 25%, a low rate compared to other studies, which indicate a rate of about 50% of patients permanently transferred to HD [45].

Out of the seven patients who were not switched on HD postoperatively, in three patients who presented an early diagnosis of parietal defects (5–7 months after the onset of PD) it was possible to temporarily interrupt the PD at 2 to 3 weeks, and in the rest of the patients, it was possible to do peritoneal exchanges with small volumes that were progressively increased until the return to the initial treatment. The decision was taken individually for each patient together with the nephrologist, depending on the clinical and biological conditions of the patients.

Many studies and guidelines (SAGES, ISPD) have attempted to establish the need for a multidisciplinary “Access Team” composed of a surgeon, nephrologist, anesthesiologist, dedicated nurse, and the implementation of local protocols and training. This ensures rapid patient access, training, and supervision both post-implantation and during other surgical events that may occur during PD [21,23]. This multidisciplinary team was established in our hospital 12 years ago and allows us to achieve high-performance results in the care and treatment of patients undergoing PD.

The study performed has limitations, being a single-center experience and retrospective, but the large number of cases included in the study reinforces the conclusions we have reached.

## 5. Conclusions

Diagnosis of ventral hernias prior to PD catheter insertion leads to a decrease in the incidence of parietal defects during PD and is mandatory in patients who are candidates for PD.

Open alloplastic surgical procedures are safe procedures with a low recurrence rate in PD patients.

The postoperative continuation of PD is feasible but the decision is to be made by the multidisciplinary team and individualized for each patient.

## Figures and Tables

**Table 1:** Type of surgical intervention.

Surgical Intervention	Number of Patients	Sex—Male	Prior Abdominal Surgery
Laparoscopic	126	64%	12
Open	7	63%	0

**Table 2:** ESRD cause, gender distribution and complications.

ESRD Diagnostic	Number of Patients	Gender	Hernia Diagnosis	Complications
Chronic glomerulonephritis	3	male	inguinal	
Chronic ischemic nephropathy	2	male	inguinal	1 Seroma
Nephrosclerosis	1	female	umbilical	
Diabetic nephropathy	1	female	umbilical	Seroma
Polycystic hepatorenal disease	1	female	umbilical	
Polycystic kidney disease	1	male	Inguinal-1 bilateral	Hematoma

**Table 3:** BMI, gender distribution, type of hernia, ESRD cause and PD duration.

Patient	Gender	ESRD Diagnostic	BMI	Type of Hernia	PD Duration (Months)
P1	M	Chronic ischemic nephropathy	22.23	Umbilical	20
P2	M	Diabetic nephropathy	29.25	Umbilical	19
P3	F	Chronic ischemic nephropathy	24.02	Umbilical	2
P4	F	Chronic glomerulonephritis	27.92	Inguinal	18
P5	M	Diabetic nephropathy	26.12	Supraumbilical	13
P6	M	Diabetic nephropathy	28.69	Umbilical	5
P7	F	Chronic glomerulonephritis	23.12	Umbilical	48
P8	F	Nephrosclerosis	23.14	Supraumbilical	8
P9	F	Chronic ischemic nephropathy	27.73	Pericatheter	21
P10	F	Chronic glomerulonephritis	26.52	Umbilical	6
P11	F	Polycystic kidney disease	31.24	Umbilical	11
P12	F	Diabetic nephropathy	30.70	Umbilical	12

**Table 4:** Number of complications and parietal defects.

Parietal Defect	No of Patients	Complications	
		Hematoma	Seroma
Inguinal	1	0	0
Umbilical	8	1	2
Supraumbilical	2	0	1
Pericatheter	1	0	1

## Data Availability

The original contributions presented in the study are included in the article, further inquiries can be directed to the corresponding authors.
